# Knowledge and perceptions of the intrauterine device among family planning providers in Nepal: a cross-sectional analysis by cadre and sector

**DOI:** 10.1186/s12913-015-0701-y

**Published:** 2015-01-28

**Authors:** Nirali M Chakraborty, Caitlin Murphy, Mahesh Paudel, Sriju Sharma

**Affiliations:** Population Services International, Washington, DC USA; Population Services International, Kathmandu, Nepal; Helen Keller International Nepal, Lalitpur, Nepal

**Keywords:** Family planning, Nurse, Auxiliary nurse midwife, IUDs, Franchise, Public sector, Nepal, Task sharing

## Abstract

**Background:**

Nepal has high unmet need for family planning and low use of intrauterine devices (IUDs). While clients’ attitudes toward the IUD are known in a variety of contexts, little is known about providers’ knowledge and perceptions of the IUD in developing countries. Nepal’s liberal IUD service provision policies allow the opportunity to explore provider knowledge and perceptions across cadres and sectors. This research contributes to an understanding of providers’ IUD perceptions in low-resource environments, and increases evidence for IUD task-sharing and private sector involvement.

**Methods:**

A questionnaire was administered to 345 nurses and auxiliary nurse midwives (ANMs) affiliated with the private *Mahila Swastha Sewa (MSS)* franchise, public sector, or private non-franchise sector. All providers had been trained in TCu 380A IUD insertion and removal. The questionnaire captured providers’ IUD experience, knowledge, and perceived barriers to recommendation. Descriptive, multivariate linear, and multinomial logistic regression was conducted, comparing providers between cadre and sector.

**Results:**

On average, providers answered 21.5 of 35 questions correctly, for a score of 61.4%. Providers scored the lowest on IUD medical eligibility, answering 5.9 of 14 questions correctly. Over 50% of providers were able to name the four side effects most frequently associated with the IUD; however, one-third of all providers found at least one of these side effects unacceptable. Adjusted results show that cadre does not significantly impact provider’s IUD knowledge scores or side effect perceptions. Public sector affiliation was associated with higher knowledge scores regarding personal characteristic eligibility and more negative perceptions of two normal IUD side effects. IUD knowledge is significantly associated with provider’s recent training and employment at multiple facilities, and side effect perceptions are significantly associated with client volume, range of family planning methods, and region.

**Conclusions:**

Provider knowledge and attitudes towards IUD provision are similar across cadre and sector, supporting WHO task-sharing guidelines and validating Nepal’s family planning policies. However, overall provider knowledge is low. We recommend that providers need to receive further training and support to improve knowledge, manage side effects, and recognize women in periods of high unmet need - such as post-partum or post-abortion women - as suitable candidates for IUDs.

**Electronic supplementary material:**

The online version of this article (doi:10.1186/s12913-015-0701-y) contains supplementary material, which is available to authorized users.

## Background

In the context of global public health conversations around unmet need for family planning, human resources for health shortages, and task-sharing to lower-level health providers, a large number of initiatives have emerged to improve women’s access to a comprehensive mix of family planning products and services.

In Nepal, for example, the use of modern contraceptives among married women has increased from 35.4% to 43.2% over the past 10 years [1]. However, unmet need for family planning is still reported by 27.5% of married Nepalese women [1]. A low desired fertility rate (2.2) and young first birth (averaging at 20 years old) suggest the need for a sustained ability to limit or space births over several years [1]. The contraceptive methods used most frequently are sterilization (15.2% of married women), injectables (9.2%), condoms (4.3%), and oral pills (4.1%) [1]. Unintended pregnancy in Nepal is attributed to the reliance on short-term methods, which have a greater chance of method failure and discontinuation [2,3].

Research has demonstrated that long-acting methods such as intrauterine devices (IUDs) are a cost-effective and sustainable way of reducing unmet need and unintended pregnancy in low-resource settings like Nepal [2,4]. Given Nepal’s difficult topography and limited urbanization (83% of the population lives in a rural area), the IUD has been suggested as an effective and efficient method for family planning [1,3]. However, despite being one of the first modern methods introduced in Nepal, the IUD remains one of the least known and least used contraceptive methods [3]. Uptake has nearly doubled in recent years (from 0.8% in 2006 to 1.3% in 2011), but use and coverage remain low given the demonstrated demand for increased IUD accessibility [5]. Barriers to IUD access in Nepal include a lack of trained staff, limited availability of IUD supplies, limited provision of IUD services, provider bias against long-acting reversible contraception (LARC), and poor counseling skills regarding the IUD’s advantages and disadvantages [3,5].

In this context, Population Services International (PSI) has introduced a program to revitalize IUD use by training private health care providers in IUD insertion and removal, and using community health workers to educate women and dispel myths about IUDs and other family planning methods.

### Family planning provision in Nepal

National family planning services were first introduced into Nepal in 1968, but sterilization remained the primary long-term method for decades [6,7]. Following increased emphasis on contraceptive choice after the International Conference for Population and Development (ICPD), the Family Health Division (FHD) of Nepal’s Ministry of Health and Population (MoHP) developed its National Family Planning Guidelines in 1997. These guidelines were accompanied by the creation of an IUD service expansion program supported by USAID and EngenderHealth [5]. The program provided IUD training to providers at health centers and health posts, and upgraded health facilities. However, despite improvements, many health posts today remain understaffed, unmanned, and have limited hours [8].

The private sector - comprised of for-profit clinics, NGOs, and social franchise outlets - has strived to fill these IUD provision gaps. Reflective of the Nepalese government’s economic policies toward liberalization and privatization initiated in 1991, the private sector has increasingly provided a larger share of family planning services [7]. In 2011, 30% of family planning was provided outside of the public sector [1]. In support of this involvement, Nepal’s MoHP has prioritized scaling up the private health sector in their 2011-2015 Health Sector Programme Implementation Plan [9].

The Nepalese government has also attempted to fill family planning provision gaps through its progressive task-shifting policies. Starting in the 1970s, the Nepalese government created an auxiliary nurse midwife (ANM) program specifically to increase rural women’s access to healthcare. This program grew out of the recognition that Nepal’s low provider-to-client ratio necessitates that lower level cadres provide the majority of family planning services, especially in rural and remote regions [3,8,10]. Compared with nurses, who receive three or more years training and authorization to practice by a state board, ANMs receive training for 18 months in secondary school and supplementary on-the-job training [10]. ANMs are frequently the highest level of cadre in rural areas that are qualified to insert an IUD, and ANMs have been permitted to insert and remove the IUD since 1997 [3].

These policies have facilitated an environment in which Nepalese ANMs have been regularly inserting IUDs prior to the release of the World Health Organization’s 2012 task-shifting recommendations. The WHO currently recommends that nurses and midwives be permitted to insert IUDs, and suggests that more evidence is needed for ANMs [11]. Other countries in the region echo these policies: Kyrgyzstan, Pakistan, and Tajikistan all permit midwives in any sector to insert IUDs. However, globally, there are greater restrictions on the type of personnel permitted to provide IUD services. For example, in the Dominican Republic, Kazakhstan, and Laos the lowest cadre permitted to insert are doctors or gynecologists. In El Salvador, Guatemala, and Mozambique the lowest cadre permitted to insert are nurses ^a^*.* Despite years of operations research studies demonstrating the safety of task-sharing policies, hesitancy remains, perhaps due to a lack of regional evidence or comprehensive assessments of the differences between cadres [12-15].

### Providers at MSS facilities

Population Services International (PSI) supports 481 private health outlets in 49 districts in Nepal, encompassed under a branded franchise. At the time of this study, the franchise was known as *Mahila Swastha Sewa* (*MSS*), but has since been rebranded to *OK*. The facilities are independently owned and operated by physicians, nurses, or ANMs. *MSS* franchises have applied Nepal’s task-shifting policies to their approach – 88% of providers are ANMs. In addition to providing a variety of curative care services for children and adults, outlets provide maternal health and family planning products such as condoms, oral contraception, injectables, implants, and IUDs to clients.

Since 2009, PSI’s Woman’s Health Project (WHP) has sought to increase access to long-acting reversible contraception (LARC) through the *MSS* franchise, within a context of informed choice. As members of the franchise, staff are provided with training on how to counsel clients, clinical instruction on the insertion and removal of IUDs and implants, supplies and necessary equipment, quality assurance via clinical audits, supportive supervision, and assistance with demand generation. Between 2008 and 2012, 83,548 IUDs were inserted by *MSS* providers. In 2011, WHP introduced medical detailing practices to further improve *MSS* providers’ knowledge, practice, and attitudes with respect to IUDs.

Among the few studies which have explored provider knowledge about IUDs, providers demonstrate low knowledge and concerns with IUD safety, side effects, and client satisfaction [16-18]. Furthermore, only a few comparative studies between provider types have been conducted, and no studies were found that compare knowledge and perceptions of providers in private franchises versus other service delivery sectors [19].

This study quantifies provider knowledge and perceptions of IUDs in Nepal. It identifies variables that significantly correlate with providers’ IUD knowledge and perceptions, and explores differences in knowledge and side effects perceptions among two cadres (nurses vs. ANMs) and three sectors (private franchise vs. private non-franchise vs. public).

## Methods

### Study design

A cross-sectional survey was conducted between January 2012 and February 2012 among 345 providers affiliated with the private *MSS* franchise, public sector, or private non-franchise sector. *MSS* providers were randomly selected from a pool of 300 providers active in the franchise ^b^. Non-franchise providers were randomly selected from a list of public and private non-*MSS* providers in the same and neighboring districts as *MSS* providers. The eligibility criteria for inclusion was that providers have adequate facilities to provide IUD services, have at least an ANM qualification, offer FP services, and have experience in pelvic examination. Non-franchise providers were not required to own their clinic, but *MSS* providers were required to own or have a close established relationship with their clinic. The sample size was calculated to be able to detect a 15% difference (or a change of 2 correct responses) between the two groups, with 80% power and 95% confidence. A total of 176 *MSS* providers and 169 non-*MSS* providers were included in the final data set. Nine MD/OB-GYNs were excluded from analysis due to their higher cadre.

### Measurement

A questionnaire (Additional file 1) was developed following a literature search and personal communication with researchers, specialist physicians, and master trainers on IUD insertion protocols ^c^ [20-22]. General knowledge was assessed via two multiple choice and six true-false questions on TCu 380A IUD efficacy, mechanism, and the appropriate time of insertion. Knowledge of medical eligibility was assessed via 14 statements of medical history risk factors from WHO eligibility criteria (ie. anemia, obesity, STIs), [23]. Knowledge of eligibility based on personal characteristics was assessed via 13 demographic statements (ie. poor, nulliparious, illiteracy, unmarried). While we refer to this category as a measurement of “knowledge”, it also reveals provider attitudes: international medical guidelines indicate that there is no medical reason why an otherwise healthy woman with the characteristics described would not be eligible for an IUD. Perceptions of IUD side effects were recorded via an open-response question that asked providers to name all of the side effects and adverse events they associate with the TCu 380A IUD (ie. excessive bleeding, cramping, risk of ectopic pregnancy). Follow-up questions probed whether providers considered each side effect acceptable or unacceptable. As side effects are cited as a major source of IUD discontinuation [24], understanding provider knowledge, attitudes, and willingness to manage them is desirable.

Questionnaires were administered in Nepali by a trained interviewer over a period of six weeks in each provider’s facility. The questionnaire took about 50 minutes to administer. Ethical approval to conduct the study was received from the Nepal Health Research Council and the PSI Research Ethics Board. All providers were consented prior to questionnaire administration.

Equally weighted summative scores were generated for each of three knowledge sub-categories (general knowledge, medical eligibility knowledge, and personal characteristic eligibility knowledge), and were reported both as a raw score and an average of the proportion of correct responses. Responses regarding the four side effects known to be medically manageable (excessive bleeding, painful menstruation, cramping, and spotting) were divided into three categories: providers who did not name the side effect, providers who named it but did not say that it would prevent them from recommending the IUD (“acceptable”), and providers who named a side effect and said that it would prevent them from recommending the IUD (“unacceptable”).

### Covariate selection

Covariates were chosen based on attributes demonstrated to be associated with provider knowledge and perceptions of health service scenarios. A literature review revealed *experience* as one of the strongest known determinants of IUD knowledge [16]. Two frameworks were identified as sufficiently explanatory for the positive impact experience has on accurate knowledge and perceptions in health services: 1) experience produces a broader range of cases or “scripts” available for retrieval (the Stage Theory of Clinical Reasoning) and 2) experience generates understanding of the opportunities and constraints of particular clinical situations (the Dreyfus Model of Skill Acquisition) [25-28].

Covariates were chosen that can proxy for or indicate experience, and have been shown to be positively associated with family planning knowledge in previous studies. These covariates include: provider age, length of employment, volume of women served (measured through clinic volume, volume of IUDs inserted, and employment at multiple clinics), range of family planning methods offered, and past IUD training [27,29,30]. Region was chosen as a potential indicator of experience because the poor infrastructure in Nepal’s hills may result in lower client volume and resources. Under the assumption that those with financial investment in a clinic’s success may be more reluctant to support the low-cost IUD, ownership of a clinic, and sector (public vs private) were also chosen as potential indicators of experience [16,31]. Last, independent of experience, provider’s personal use of contraception was included due to its presence in previous tools that sought to capture data on IUD knowledge and perceptions [20,32].

### Analytic methods

Descriptive, multivariate linear, and multinomial logistic regression techniques were used in the analyses. Analyses were conducted on a sample of 345 providers. For multivariate analyses, the covariates chosen using the theories described above were initially included in all analyses.

Descriptive analyses compared groups using Student’s t-test and Pearson’s chi-squared test. Differences in knowledge and side effect perceptions were examined between cadres (nurses and ANMs) and sector (*MSS*, public, and private non-franchise). Frequencies were generated for all categorical variables, and differences between proportions were examined using chi-squared. Continuous variables were summarized using means and standard deviation, and differences between the means were examined using the t-test. A *p* value of <0.05 was considered statistically significant.

Backward stepwise linear regression, with probability of exclusion of p > 0.1, was used to generate one regression model for each of the three summative knowledge sub-categories and one cumulative knowledge score. Provider cadre and sector were forcibly included in each model to observe their impact.

Multinomial logistic regression was used to examine side effect responses, with the base outcome that the provider did *not* report the side effect. Models included provider socio-demographic characteristics (age, cadre, time since training) and facility characteristics (family planning client volume, methods offered, clinic location, sector). As selected covariates were dropped from the model, a likelihood ratio test was used to assess if the more parsimonious model was just as good as the full model at p < 0.05. Provider cadre and sector were forcibly included in each model. All analyses were conducted using STATA version 11 statistical software (Statacorp, College Station, TX).

## Results

### Sample characteristics

Table 1 presents the socio-demographic, facility-related, and IUD experience-related characteristics of the providers stratified by cadre. Among the 345 providers interviewed for this study, all were female and 80% were auxiliary nurse midwives. Providers were fairly similar across cadres for many characteristics: 73.0% reported that they had children, 44.6% used contraception at the time of the study, and 13.3% of providers reported that they had ever used an IUD. Nurses were significantly older than ANMs (mean age 36.8 years vs. 32.0 years, *p* < 0.001), and were less likely to work in a franchised facility (31.9% vs. 55.8%, *p* < 0.001). The majority of providers considered their facility their primary place of work (83.2%), and 29.6% owned their facility (33% among ANMs vs. 15.9% among nurses, *p* = 0.006).

**Table 1:** **Proportion and mean of provider sociodemographic characteristics and professional experience by cadre** ^**1**^

**Variable**	**ANMs n = 69**	**Nurses n = 69**	**Both cadres n = 345**
*Sociodemographic*			
Age (mean)	32.0 ± 8.79	36.8 ± 10.11***	33.0 ± 9.26
Have children	204 (73.9)	48 (69.6)	252 (73.0)
Currently using contraception	124 (44.9)	30 (43.5)	154 (44.6)
Have used IUD	35 (12.7)	11 (15.9)	46 (13.3)
*Facility-related*			
Work at MSS facility	154 (55.8)	22 (31.9)***	176 (51.0)
Years employed at facility (mean)	5.6 ± 5.68	6.5 ± 6.54	5.8 ± 5.86
Owns facility	91 (33.0)	11 (15.9)**	102 (29.6)
Facility is primary place of work	229 (83.0)	58 (84.1)	287 (83.2)
Facility is located in hill region	112 (40.6)	32 (46.4)	144 (41.7)
Number of FP clients per week (mean)	23.1 ± 19.21	33.1 ± 32.71***	25.1 ± 22.84
Number of FP methods offered at facility (mean)	6.2 ± 1.46	7.5 ± 1.47***	6.5 ± 1.54
Facility offers IUDs	245 (88.8)	69 (100.0)**	314 (91.0)
*IUD experience*			
Have inserted IUD < 6 months ago	246 (89.1)	62 (89.9)	308 (89.3)
Number of IUDs inserted in last 6 months (mean)	24.7 ± 27.82	32.8 ± 39.21**	26.3 ± 30.55
Years since most recent IUD training (mean)	2.1 ± 3.22	4.4 ± 4.89***	2.6 ± 3.73

^1^Ttests and chi-squared tests were used to test the differences between cadre. Differences are indicated by **p < 0.05 and ***p < 0.001. Values are given as mean ± SD or number (percentage).

The family planning profile of facilities and providers’ IUD experiences differed by cadre. Nurses’ facilities offered more family planning methods (μ = 7.5 methods, SD = 1.47 vs. μ = 6.2 methods, SD = 1.47), and more of their facilities offered IUDs (100% vs. 88.8%). Although all providers had to offer IUDs to be eligible for the study, only 91% reported having IUDs available at the time of the survey, due to stock-outs. Nurses had inserted more IUDs in the past 6 months than ANMs (μ = 32.8 IUDs, SD = 39.21 vs. μ = 24.7 IUDs, SD = 27.82), but nurses’ facilities also saw more family planning clients per week (μ = 33.1 clients, SD = 32.71 vs. μ = 23.1 clients, SD = 19.21). Nurses received their current IUD training less recently (μ = 4.4 years, SD = 4.89 vs. μ = 2.1 years, SD = 3.22).

Fifty-one percent of all providers belonged to a *MSS* franchise. The remaining providers worked in the public sector (35.6%) and private non-franchise facilities (13.6%). The demographic differences between providers in these three sectors are shown in Additional file 2.

### Providers’ knowledge scores

Providers answered an average of 61.4% of all knowledge questions correctly (Table 2). Knowledge varied between the three knowledge sub-categories.

**Table 2:** **Provider knowledge questions and proportion of correct responses**

**Question**	**Correct response**
	**n (%)**
	**n = 345**
**General knowledge of the IUD**	
What do researchers believe is the main mechanism of copper-bearing IUDs at preventing pregnancy?	309 (89.6)
How effective is the widely-used copper TCu 380A IUD at preventing pregnancy annually?	166 (48.1)
When can the IUD be safely inserted, provided it is reasonably certain the woman is not pregnant?	
Anytime during the menstrual cycle, provided the woman has no signs of infection	312 (90.4)
6 months after delivery	293 (84.9)
When a woman is menstruating	287 (83.2)
Four weeks after delivery	271 (78.6)
Within 48 hours post-partum, provided there is no infection or hemorrhage	230 (66.7)
Up to 7 days post-partum, provided there is no infection or hemorrhage	136 (39.4)
**Medical eligibility knowledge for the IUD**	
When is a woman medically eligible, eligible but with some screening or caution, or not medically eligible for the TCU 380A IUD?	
Smokes, < 15 cigarettes per day	241 (69.9)
Overweight/obese	238 (69.0)
Recently undergone first trimester abortion	201 (58.3)
Currently breastfeeding	193 (55.9)
Hypertension patient	182 (52.8)
Vaginal discharge	154 (44.6)
Irregular menstrual pattern	129 (37.4)
On Anti-Retroviral Therapy (clinically well)	127 (36.8)
HIV positive	124 (35.9)
Had pelvic inflammatory disease 3 years ago	115 (33.3)
Current STI patient	112 (32.5)
Less than 48 hours post-partum	109 (31.6)
Iron-deficiency anemia	108 (31.3)
History of ectopic pregnancy	4 (1.2)
**Personal characteristic eligibility for the IUD**	
Would you recommend the TCu 380A IUD to this woman as a method, not recommend, or are unsure?	
A woman who wants to delay her pregnancy	344 (99.7)
A woman who is illiterate	344 (99.7)
A woman who is very poor	344 (99.7)
A woman who has one child	339 (98.3)
A woman who does not want to have any more children	338 (98.0)
A woman who has four children	334 (96.8)
A woman who does heavy physical labor every day	302 (87.5)
A woman who is of very small stature (short, tiny, etc)	278 (80.6)
A woman who is 17 years old	207 (60.0)
A woman who has no children (nulliparious)	166 (48.1)
A woman who is not married	162 (47.0)
A woman who has more than one sexual partner	136 (39.4)
A woman whose sexual partner is not monogamous	98 (28.4)

#### General knowledge

Knowledge of general IUD properties and mechanisms was fair. Providers answered an average of 72.5% questions correctly (5.8 questions out of 8.) The majority of providers knew the main mechanism of action of the TCu 380A IUD (89.6%), and over three-fourths knew that the IUD can be safely inserted when a woman is menstruating, any time during the menstrual cycle, four weeks after giving birth, and six months after giving birth (83.4%, 90.4%, 78.6%, and 84.9%, respectively). Two-thirds of providers knew that the IUD can be safely inserted when a woman is 48 hours post-partum (66.7%). However, only 39.4% of providers knew that the IUD *cannot* be inserted between one week and 4 weeks post-partum. Additionally, less than half knew how effective the IUD is at preventing pregnancy (48.1%).

#### Medical eligibility

Knowledge of a woman’s medical eligibility for IUD insertion was poor. Providers were correct regarding a woman’s eligibility for the device for an average of 5.9 out of 14 cases (42.1% correct). For 9 of 14 cases, less than 50% of providers correctly identified the conditions under which a woman would be eligible for an IUD. In 5 of these 9 cases, less than 40% providers correctly identified that women were eligible for the IUD *without* screening: history of ectopic pregnancy (1.2%), anemia (31.3%), pelvic inflammatory disease 3 years ago (33.3%), less than 48 hours post-partum (31.6%), and irregular menstruation (37.4%). In 4 of these 9 cases, less than 50% of providers correctly identified that women were eligible for the IUD *with* screening: STI patient (32.5%), HIV positive (35.9%), antiretroviral therapy use (36.8%), and vaginal discharge (44.6%).

#### Personal characteristic eligibility knowledge

Knowledge of personal characteristic eligibility was good. Providers were correct regarding a woman’s eligibility for the IUD for an average of 9.8 of 13 cases (75.4% correct). For 8 cases, over 80% of providers correctly identified that a woman was eligible for IUD insertion: being of small stature (80.6%), doing physical labor every day (87.5%), having one child (98.3%), having four children (96.8%), not wanting to have any more children (97.7%), wanting to delay her next pregnancy (99.7%), being illiterate (99.7%), and being very poor (99.7%). While three-fifths of providers considered a woman who is 17 years old eligible for an IUD (60%), less than 50% of providers considered an unmarried woman or a woman without children eligible (47.0% and 48.1%, respectively). Last, less than 50% of providers correctly identified the two cases that should *limit* a provider’s recommendation of exclusive IUD use: having a non-monogamous partner (28.4%) and having multiple partners (39.4%).

### Factors associated with knowledge scores

Bivariate analysis was conducted using the summative scores for each knowledge sub-category (general knowledge, medical eligibility knowledge, personal characteristic eligibility knowledge) and their cumulative score (overall knowledge). When each knowledge category was compared by cadre, no significant differences in scores were indicated between ANMs and nurses.

**Figure 1:**
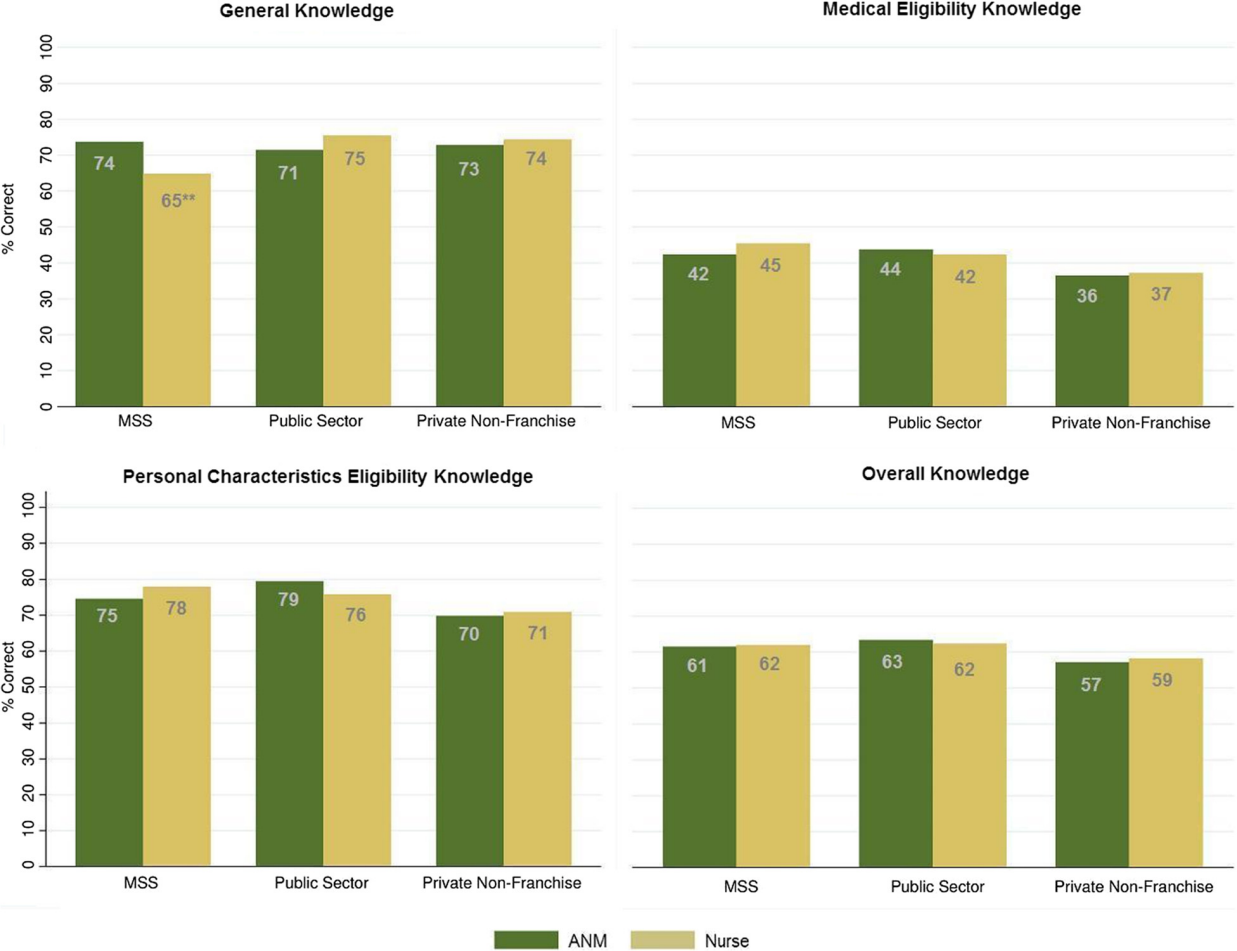
**Proportion of correct responses for knowledge sub-categories, by cadre and sector.** Significant differences within each sector are indicated by **p <0.05.

Tabulating each score by sector indicated that there were significant differences between public sector and private non-franchise providers in two knowledge categories. Public sector providers had significantly higher personal characteristic eligibility scores and overall knowledge scores than private non-franchise providers (78.5% vs. 70.2%, *p* < 0.001 and 63.1% vs. 57.6%, *p* = 0.016). No significant differences in knowledge scores emerged between public sector and franchise providers. When cadres were compared within each sector, only one significant difference emerged: *MSS* ANMs have significantly higher general knowledge scores than *MSS* nurses (73.8% vs. 65.0%, *p* = 0.007) (See Figure 1).

Results from stepwise linear regression analysis of the factors associated with IUD knowledge are presented in Table 3. After adjusting for several sociodemographic, facility-related, and IUD experience-related characteristics, five covariates were no longer significantly associated with IUD knowledge: provider age, years of employment at facility, ownership, the volume of FP clients per week, and the number of IUDs the provider inserted in the last 6 months. Two covariates remained significantly associated with three of the four knowledge scores: recent receipt of IUD training and employment at multiple facilities.

**Table 3:** **Multivariate linear regression of factors associated with each knowledge subcategory score, n = 340** ^**1**^

**Variable**		**General knowledge score**	**Medical eligibility score**	**Personal characteristic eligibility score**	**Overall knowledge score**
		**ß coefficient**	**ß coefficient**	**ß coefficient**	**ß coefficient**
		**(95% CI)**	**(95% CI)**	**(95% CI)**	**(95% CI)**
*Cadre*					
	Provider is Nurse	1.00	1.00	1.00	1.00
	Provider is ANM	−0.034	0.078	−0.156	−0.127
		(-0.35 to 0.29)	(-0.67 to 0.82)	(-0.61 to 0.30)	(-1.23 to 0.98)
*Sector*					
	Provider works at MSS facility	1.00	1.00	1.00	1.00
	Provider works in public sector	−0.235	0.436	0.622***	0.833
		(-0.52 to 0.05)	(-0.27 to 1.15)	(0.19 to 1.05)	(-0.20 to 1.87)
	Provider works in private non-franchise	−0.238	−0.389	−0.522	−1.125
		(-0.64 to 0.16)	(-1.30 to 0.52)	(-1.08 to 0.04)	(-2.42 to 0.18)
*Facility characteristics*					
	Provider currently uses contraception	0.215		0.299	0.895**
		(-0.04 to 0.47)		(-0.05 to 0.65)	(0.08 to 1.71)
	Facility is provider’s primary place of work		−0.847**	−0.528**	−1.306**
			(-1.58 to -0.11)	(-0.99 to -0.06)	(-2.40 to -0.21)
	# of FP methods facility offers		0.252**		0.278
			(0.05 to 0.45)		(-0.02 to 0.57)
	Facility located in hill region	−0.767***	0.702**		
		(-1.03 to -0.51)	(0.12 to 1.28)		
	Years since provider’s most recent IUD training		−0.110**	−0.059**	−0.19***
			(-0.19 to -0.03)	(-0.11 to 0.00)	(-0.32 to -0.06)

^**1**^Sample size reflects effective sample size in full model. Statistical significance is indicated by **p < 0.05 and ***p < 0.001.

Other covariates’ importance varied between the four knowledge categories. Providers with facilities located in the hill region had significantly lower general knowledge scores and higher medical eligibility scores. (Adj. β = -0.767, *p* = <0.001, 95% CI: -1.03 to -0.51 and Adj. β = 0.702, *p* = 0.018, 95% CI: 0.12–1.28); wider ranges of family planning methods were significantly associated with higher medical eligibility scores (Adj. β = 0.252, *p* = 0.014, 95% CI: 0.05–0.45); and providers using contraception had significantly higher overall knowledge scores (Adj. β = 0.895, *p* = 0.032, 95% CI: 0.08–1.71).

Adjusted results show that provider’s knowledge is not correlated with the provider’s position as a nurse or ANM. Similarly, for three of four knowledge categories, sector is not associated with knowledge. For personal characteristic eligibility knowledge, public sector providers have significantly higher scores than franchised providers (Adj. β = 0.622, *p* = 0.005, 95% CI: 0.19–1.05).

### Providers’ side effect perceptions

When providers were asked to list side effects or adverse events they associate with the IUD, over 50% of providers named the four side effects shown in Table 4. For each side effect mentioned, providers were further asked to respond to whether they found it unacceptable, leading them to not recommend the IUD. Overall, providers named an average of 4.3 side effects and found an average of 1.7 side effects unacceptable. Among the four side effects listed in Table 4, 34.2% of all providers found the common side effect of excessive bleeding unacceptable, and over 31.3% of providers reported that the rare side effect of spotting was unacceptable.

**Table 4:** **Perceptions of IUD side effects among all providers, n = 345**

**Side effect**	**Did not name side effect**	**Named side effect & considers it acceptable**	**Named side effect & considers it unacceptable**
	**n (%)**	**n (%)**	**n (%)**
Excessive bleeding	106 (30.7)	121 (35.1)	118 (34.2)
Painful menstruation	138 (40.0)	139 (40.3)	68 (19.7)
Cramping	93 (27.0)	174 (50.4)	78 (22.6)
Spotting	50 (14.5)	187 (54.2)	108 (31.3)

Lower percentages of providers named occurrences which are medically uncommon, or not associated with the IUD (data not shown). These occurrences included ectopic pregnancy (26.1%), acquisition of HIV (15.4%), acquiring another STI (17.7%), nausea (19.4%), weight gain (8.1%), and headaches (12.5%).

### Factors associated with side effect perceptions

A multinomial logistic regression model was used to calculate the relative risk of a provider naming a side effect and considering it acceptable, or naming it and considering it unacceptable, as compared to not naming it at all. As with the knowledge analyses, models were intended to assess if provider cadre or sector had any significant association with the perception of the side effects. Analyses concentrate on the side effects providers mentioned most frequently in Table 4.

Adjusted results (Tables 5 and 6) show that there is no difference between nurses and auxiliary nurse midwives with regard to the probability that they will name any of the four side effects acceptable or unacceptable. The sector in which a provider works, however, does impact side effect perceptions. As compared to franchised providers, public sector providers are twice as likely to name excessive bleeding unacceptable than not name it at all (*p* = 0.024, 95% CI = 1.10 –3.82), and twice as likely to name painful menstruation unacceptable than not at all (*p* = .024, 95% CI = 1.04–4.37). Private non-franchise providers are significantly less likely than franchised providers to name spotting unacceptable than not at all (RRR = 0.290, *p* =0.019, 95% CI: 0.10–0.82). Last, private non-franchise providers are less likely than franchise providers to name cramping or spotting side effects *acceptable* as compared to not at all (RRR = 0.304, *p* = 0.004, 95% CI: 0.14–0.68 and RRR = 0.403, *p* = 0.043, 95% CI: 0.17–0.97).

**Table 5:** **Adjusted relative risk ratio (RRR) of perceptions of IUD side effects: excessive bleeding & painful menstruation** ^**1**^

**Variable**	**Excessive bleeding**		**Painful menstruation**	
	**Acceptable**	**Unacceptable**	**Acceptable**	**Unacceptable**
	**RRR**	**RRR**	**RRR**	**RRR**
	**(95% CI)**	**(95% CI)**	**(95% CI)**	**(95% CI)**
	**n = 121**	**n = 118**	**n = 139**	**n = 68**
*Cadre*				
Provider is Nurse	1.00	1.00	1.00	1.00
Provider is ANM	1.027	1.468	0.615	1.151
	(0.52 to 2.02)	(0.72 to 3.02)	(0.32 to 1.18)	(0.47 to 2.80)
*Sector*				
Provider works at MSS facility	1.00	1.00	1.00	1.00
Provider works in public sector	1.432	2.052**	0.991	2.127**
	(0.77 to 2.66)	(1.10 to 3.82)	(0.54 to 1.82)	(1.04 to 4.37)
Provider works in private non-franchise	1.966	2.138	0.565	0.925
	(0.83 to 4.68)	(0.87 to 5.27)	(0.75 to 1.26)	(0.33 to 2.56)
*Facility characteristics*				
# of FP methods facility offers			1.246**	0.937
			(1.04 to 1.50)	(0.76 to 1.16)
# of FP patients/week	0.987***	0.979***	0.998	0.983
	(0.97 to 1.00)	(0.96 to 0.99)	(0.99 to 1.00)	(0.97 to 1.00)
Facility located in hill region			0.554**	0.988
			(0.33 to 0.94)	(0.53 to 1.84)

^1^As compared to not reporting the side effect at all. Statistical significance is indicated by **p < 0.05 and ***p < 0.001.

**Table 6:** **Adjusted relative risk ratio (RRR) of perceptions of IUD side effects: cramping & spotting** ^**1**^

**Variable**	**Cramping**		**Spotting**	
	**Acceptable**	**Unacceptable**	**Acceptable**	**Unacceptable**
	**RRR**	**RRR**	**RRR**	**RRR**
	**(95% CI)**	**(95% CI)**	**(95% CI)**	**(95% CI)**
	**n = 174**	**n = 78**	**n = 187**	**n = 108**
*Cadre*				
Provider is nurse	1.00	1.00	1.00	1.00
Provider is ANM	1.175	1.703	0.480	0.693
	(0.61 to 2.27)	(0.69 to 4.18)	(0.20 to 1.18)	(0.25 to 1.91)
*Sector*				
Provider works at MSS facility	1.00	1.00	1.00	1.00
Provider works in public sector	0.663	1.374	0.908	1.079
	(0.35 to 1.25)	(0.66 to 2.86)	(0.43 to 1.94)	(0.48 to 2.42)
Provider works in private non-franchise	0.304***	0.410	0.403**	0.290**
	(0.14 to 0.68)	(0.14 to 1.22)	(0.17 to 0.97)	(0.10 to 0.82)
*Facility characteristics*				
# of FP methods facility offers	1.142	0.747**	1.017	0.781
	(0.94 to 1.39)	(0.59 to 0.94)	(0.81 to 1.28)	(0.61 to 1.00)
# of FP patients/week				
Facility located in hill region	0.547**	1.255		
	(0.31 to 0.96)	(0.65 to 2.42)		

^1^As compared to not reporting the side effect at all. Statistical significance is indicated by **p < 0.05 and ***p < 0.001.

As the number of family planning clients increases, providers are less likely to report excessive bleeding as a side effect (RRR = 0.987, *p* = 0.032, 95% CI: 0.97–1.00) and less likely to report it as an unacceptable barrier than not report it at all (RRR = 0.979, *p* = 0.003, 95% CI: 0.96–0.99). Providers at facilities with a larger range of FP methods are less likely to report cramping as an unacceptable side effect (RRR = 0.747, *p* = 0.013, 95% CI: 0.59–0.94), and more likely to report painful menstruation as an acceptable side effect (RRR = 1.246, *p* = 0.019, 95% CI: 1.04–1.50). Results also showed that providers employed at facilities in Nepal’s hill region are significantly less likely to name painful menstruation and cramping as side effects of the IUD (RRR = 0.554, *p* = 0.027, 95% CI: 0.33–0.94 and RRR = 0.547, *p* = 0.034, 95% CI: 0.31–0.96, respectively).

**Figure 2:**
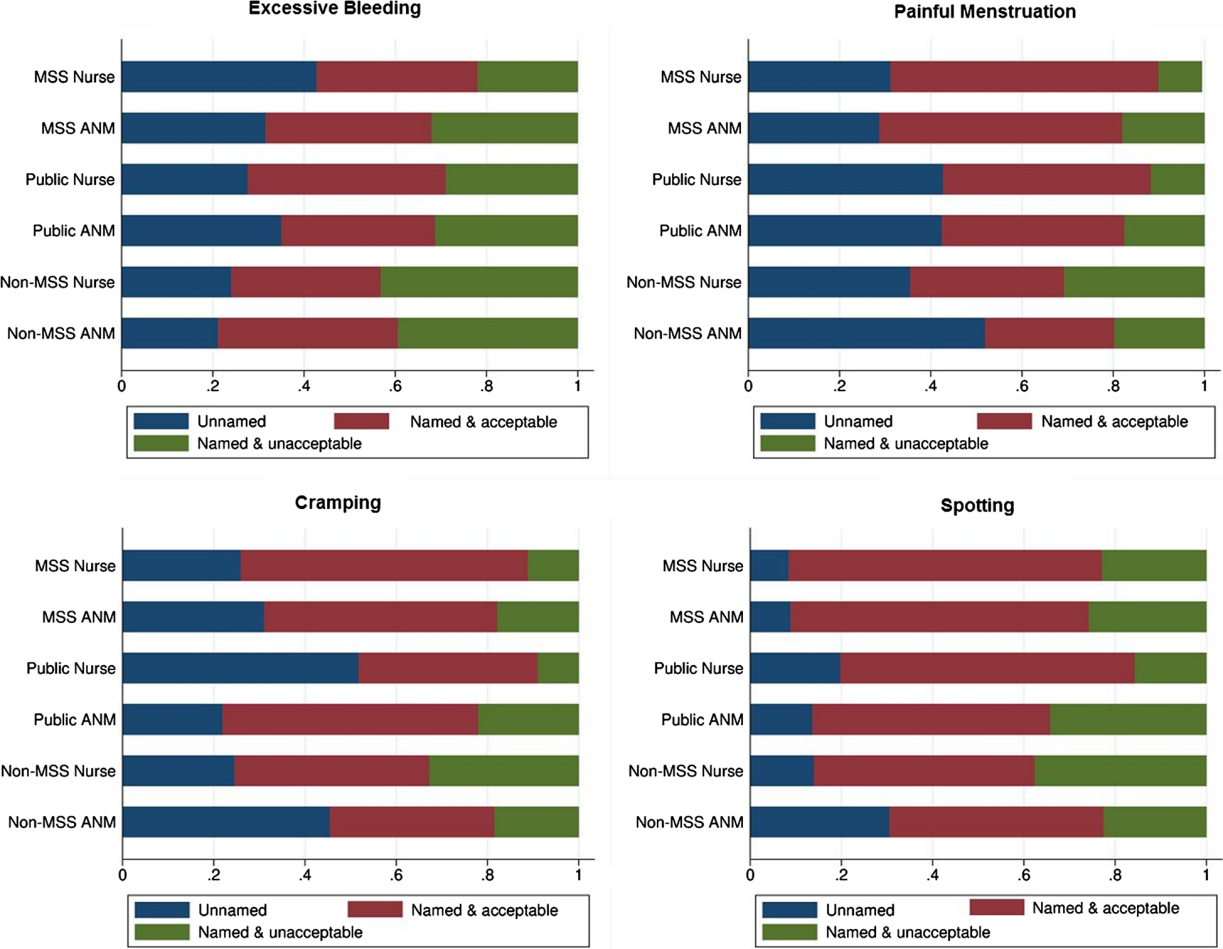
**Predicted probability of perceived IUD side effects, by cadre and sector.** Each figure is adjusted for all covariates in Tables 5 and 6. “Non-MSS Nurse” and “Non-MSS ANM” refer to private non-franchise providers.

Figure 2 visualizes the predicted probabilities of each of the three outcomes (not naming the side effect, naming it and considering it acceptable, naming it and considering it unacceptable) for nurses and ANMs from the three types of facilities in the sample. Probabilities are adjusted for all other variables in the model.

## Discussion

This study, and the subsequent analyses, had several purposes. First, we attempted to establish a baseline of provider knowledge, attitudes, and perceptions regarding IUDs in Nepal. Second, we compared franchised providers, with whom PSI works on a regular and ongoing basis, with providers who work in the public sector and other independent private providers who do not receive PSI support. Finally, given the WHO guidelines for task-sharing and the desire for more evidence on the capabilities of lower level health workers, we compared nurses with auxiliary nurse midwives on knowledge, attitudes, and perceptions of IUD related side effects.

As an international organization promoting IUD service delivery in 20 countries, PSI has developed a system used to train providers on IUDs. Results from this study indicate that provider knowledge of the IUD’s mechanism and timing of insertion are generally high, with providers scoring over 70% on the combination of these questions. Nevertheless, a more detailed analysis of responses on general knowledge indicates that 60% of providers incorrectly believe that an IUD can be inserted up to 7 days post-partum. More concerning, providers’ knowledge of which types of women can be eligible for the IUD is low, with less than 50% of providers responding correctly on several items. The overall curriculum on medical eligibility for IUDs should be examined, revised where appropriate, or more emphasis should be placed on certain types of women who may have an unmet need in the Nepalese context - such as women who are currently breastfeeding, are anemic, immediately post-partum, or have undergone a recent first trimester abortion.

The results from this study correspond to studies in Turkey, Philippines, Guatemala and Honduras regarding successful provision or comparable findings between ANMs and higher cadre health workers, [12,14,15,17], and is a positive finding for Nepal. Findings also indicate that the range of methods provided at a facility, which can be a proxy for the size and sophistication of the facility, is significantly associated with provider knowledge, similar to studies on emergency contraception in Ghana, general contraceptive provision in the United States, and others [29,30]. In contrast to a recent study in Pakistan, which found that a provider’s lifetime experience with IUDs is a strong predictor of knowledge, we did not find a relationship between IUD insertion experience (as measured in our study) and knowledge [16].

While the public sector was observed to have significantly higher personal characteristic eligibility knowledge scores, scores among all providers in this category were already fairly high overall. These findings present evidence that private providers share similar levels of competence as public providers. Evidence of equal competence between sectors is timely, given the rise of the private sector in family planning provision in Nepal and the Ministry of Health’s recent prioritization of public-private health partnerships. This suggests that private providers can help fill IUD provision gaps in Nepal that result from challenges at lower-level public Health Posts.

Multivariate analysis revealed two covariates to be consistently associated with provider knowledge across the knowledge categories. This included recent receipt of IUD training and simultaneous employment at multiple clinics (indicated by the negative association between knowledge and a facility being the provider’s primary place of work). This evidence supports the provision of refresher trainings or other methods to improve knowledge. Additionally, it backs theories that the increased experience provided by exposure to a wider range of clients may facilitate increased knowledge [25-28]. Routine quality control and supportive supervision visits, as provided to PSI’s franchised providers, can be used to further improve knowledge and attitudes among some providers, and applies theories of provider behavior change to influence providers to become advocates for a wider range of contraceptive options. This approach, while resource intensive, may be more effective than training sessions alone and may be better received by providers [33-35]. Spill-over into public sector providers may occur, as providers appear to have multiple sources of employment. However, public sector providers are less likely to have access to contraceptive technology updates or ongoing correction of misperceptions.

The provider behavior change approach becomes increasingly attractive when considering how to address providers’ ability to appropriately identify and address the side effects associated with IUDs. Providers in this study demonstrated a poor understanding of which side effects are routinely associated with IUDs; for example, 85% of providers named the clinically rare side effect of spotting, indicating that it may be unduly stressed in previous training. Further, our finding that between 20% and 35% of providers consider the common side effects of painful menstruation, cramping, and excessive bleeding unacceptable suggests that providers may not have the skills and tools needed to help clients manage these side effects. While no differences between cadres emerged, differences between sectors were present: public sector providers were twice as likely to consider excessive bleeding and painful menstruation to be unacceptable barriers to IUD provision. These side effects can be addressed by counseling women to be prepared with clean cloths and sanitary pads, and by prescribing readily available and affordable pain medication like Ibuprofen. Interestingly, providers located in the more remote hill regions of Nepal appeared less concerned with side effects. This may be a function of the fact that the benefits of an IUD, which does not require future visits to a health facility, are significantly more pronounced in a rural and remote terrain. Despite the association between recent training and factual knowledge, there was no association between training and perceptions of side effects – a further reason to approach this barrier in other ways, such as supportive supervision.

This study is unique in its comprehensive approach to assessing provider knowledge, attitudes, and perceptions with regard to IUDs. The questionnaire, informed by numerous studies, discussions with OB/GYN teaching faculty, and master trainers for PSI’s interventions, captured aspects – such as biases in IUD provision due to the personal characteristics of the patient – that have not been captured systematically in other settings. Nevertheless, the study is limited because of its cross-sectional nature; provider characteristics, the sector in which they work, and training level may be endogenous variables. Additionally, only 20% of the sampled providers were nurses, which may lead to some comparisons being underpowered to detect significant differences. The descriptive analyses of differences between cadres (Table 1) and sectors (Additional file 2) indicate that nurses are older, work at higher volume facilities with greater method mix, and are less likely to work at a franchised facility. Those who work at public sector facilities are also older and see more clients per week, but do not insert as many IUDs as those in the franchises. Variables capturing these differences were included in all multivariate models, to ensure they did not confound results; however, some variables like age and IUD volume were not significantly associated with any of the eight outcomes presented in this paper. Given the analytic technique used, variables with no association, although thought to be of theoretical importance, are not presented in final models.

Although considered a baseline study, it is important to note that nearly all providers have received some type of intervention, whether it be a training or participation in the PSI franchise and its accompanying services: supervision, training, and commodity security. It is possible that the lack of difference seen between cadres and between sectors is a consequence of the higher number of ANMs in the franchise sector, who have received a more intensive intervention. In other contexts, these findings can be tested by assessing provider attitudes prior to their participation in a franchise, and by following providers longitudinally. The lack of variation in knowledge (see Figure 1) is noteworthy, and should be further examined in other studies, and particularly with a wider range of cadres eligible to insert IUDs (such as general physicians, and OB/GYNs). In Nepal, franchised providers will continue to receive a more intensive intervention, including continued routine assessment of their clinical abilities. A later assessment of their knowledge and attitudes could also indicate if this intervention is having the intended effect.

## Conclusions

Despite WHO guidelines stating that ANMs be allowed to insert IUDs, many countries in the region do not allow or practice task-sharing, including India, Laos, and Myanmar. These results can be used to help advocate for more liberal policies for IUD insertion in these neighboring countries, which have a high burden of unmet need for contraception and where women would benefit from a low-cost long-acting method.

A variety of logistical constraints limited the assessment of providers to a questionnaire, which, notably, does not assess their clinical skills. Based on the lack of difference between cadre, we conclude that lower-level cadres, whom the WHO recommends be allowed to provide IUD services, are equally able to provide the services. However, public sector providers have a supervisory support system in many facilities, and those in the PSI franchise network receive routine clinical support, leading authors to feel more confident in their clinical abilities. The clinical skills of independent private providers are less well known. Future research should be conducted to compare clinical skills of providers, by cadre and sector.

For Nepal, and other contexts where IUD provision by lower-level health workers is permitted, programmatic efforts to increase the use of IUDs must consider provider perceptions, barriers, and motivations. Despite training providers, our findings indicated that provider knowledge, particularly with regard to medical eligibility for the IUD, was low. The concept of training could be expanded to reduce potential barriers to IUD use. This includes addressing IUDs in pre-service curricula, on-the-job training opportunities, provider peer-to-peer education, satisfied client advocacy, and continued evidence that IUDs can be used safely by a variety of women.

## Endnotes

^a^As per personal communication with local PSI staff: Loddys Abreu, Aleya Ali, Sofia Bandomia, Julia Gall, Sarah Romorini, and Karen Steele.

^b^At the time of this study, PSI defined providers as “active” if they had received training and inserted an IUD in the previous three months.

^c^This included personal communication with Dr. Paul Blumenthal of SPIRES/Stanford University School of Medicine; Dr. Eve Espey of the University of New Mexico, Albuquerque; Dr. Chelsea Polis of USAID; Dr. David Hubacher of FHI 360; & Dr. Amy Voedisch of Stanford University School of Medicine.

## Additional files

Additional file 1:
**Provider IUD knowledge questionnaire.**
Additional file 2:
**Proportions and mean of provider sociodemographic characteristics & professional experience, by sector.**

## Abbreviations

ANM: Auxiliary nurse midwife
IUD: Intrauterine device
MSS: Mahila Swastha Sewa franchise
PSI: Population Services International
