# Supplementary material for: Knowledge and perceptions of the intrauterine device among family planning providers in Nepal: a cross-sectional analysis by cadre and sector
Source: BMC Health Serv Res. 2015 Jan 28;15:39. doi: 10.1186/s12913-015-0701-y (PMC4322443; doi:10.1186/s12913-015-0701-y)
Supplement: Additional file 1: — Provider IUD knowledge questionnaire. [file 12913_2015_701_MOESM1_ESM.pdf]

Provider IUD Knowledge Questionnaire

Nirali M. Chakraborty, Ph.D.
Population Services International

Note: Questionnaire designed for PSI service delivery context. Some questionnaire responses are meant to be adjusted to reflect the country context. Relevant instructions are provided in survey manual to PSI staff. Please credit PSI and author in reproductions or use of any or all of this questionnaire.

NO.	QUESTIONS	RESPONSES	CODE		SKIP
**Q109**	Do you have any children?	Yes		1	
		No		0	→ Q111
**Q110**	How many children do you have?	|__|__| children			
**Q111**	Are you and your sexual partner currently using any contraceptive method to prevent pregnancy?<br><br>*IF CLIENT HAS NO SEXUAL PARTNER, SAME SEX PARTNER, OR IS MEDICALLY UNABLE TO CONCEIVE (INFERTILE, PAST REPRODUCTIVE AGE), MARK N/A.*	Yes		1	
		No		0	→ Q113
		N/A		98	→ Q113
**Q112**	Which method are you and your partner currently using to prevent pregnancy?<br><br>*READ ANSWERS AND ALLOW FOR MULTIPLE RESPONSES.*		**YES**	**NO**	
		A. Pill	1	0	
		B. Injectable	1	0	
		C. Condoms	1	0	
		D. IUD	1	0	
		E. Implant	1	0	
		F. Female sterilization	1	0	
		G. Male sterilization	1	0	
		H. Withdrawal	1	0	
		I. Lactational amenorrhea	1	0	
		J. Periodic abstinence	1	0	
		K. Other ______________________<br>*SPECIFY*	1	1	
**Q113**	Have you ever used an IUD?<br><br>*IF THE CLIENT IS MALE, MARK N/A.*	Yes		1	
		No		0	→ End section
		N/A		98	→ End section
**Q114**	Has your current sexual partner ever used an IUD?<br><br>*IF THE CLIENT IS FEMALE, MARK N/A.*	Yes		1	
		No		0	→ End section
		N/A		98	→ End section

PROVIDER CHARACTERISTICS

READ TO PROVIDER: ***Next, I would like to ask you some questions about your experience at this clinic.***

CLINIC PROFILE

NO.	QUESTIONS	RESPONSES	CODE		
**Q115**	What services are offered at this clinic?<br><br>*READ ANSWERS AND ALLOW FOR MULTIPLE RESPONSES.*		**YES**	**NO**	
		A. Family Planning	1	0	
		B. Antenatal Care	1	0	
		C. Labor and Delivery Care	1	0	
		D. Postnatal Care	1	0	
		E. STI diagnosis/treatment	1	0	
		F. HIV/AIDS care or treatment	1	0	
		G. Child health	1	0	
		H. Abortion services	1	0	
**Q116**	What services do you personally provide at this clinic?<br><br>*CHECK ANSWERS TO QUESTION 115. IF SERVICES ARE NOT OFFERED, MARK N/A.*<br>*FOR SERVICES OFFERED, READ ANSWERS AND ALLOW FOR MULTIPLE RESPONSES.*		**YES**	**NO**	**N/A**
		A. Family Planning	1	0	98
		B. Antenatal Care	1	0	98
		C. Labor and Delivery Care	1	0	98
		D. Postnatal Care	1	0	98
		E. STI diagnosis/treatment	1	0	98
		F. HIV/AIDS care or treatment	1	0	98
		G. Child health	1	0	98
		H. Abortion services	1	0	98
**Q117**	What family planning methods do you offer at this clinic?<br><br>*READ ANSWERS AND ALLOW FOR MULTIPLE RESPONSES.*		**YES**	**NO**	
		A. Oral contraceptive	1	0	
		B. Injectable/Depo	1	0	
		C. Condoms	1	0	
		D. IUD	1	0	
		E. Implant	1	0	
		F. Female sterilization	1	0	
		G. Male sterilization	1	0	
		H. Fertility awareness methods	1	0	
		I. Emergency contraception	1	0	
		J. Other ___________________<br>*SPECIFY*	1	0	
**Q118**	Is this method currently in stock?<br><br>*CHECK ANSWERS TO QUESTION Q117. IF METHODS ARE NOT OFFERED, MARK N/A.*<br>*FOR METHODS OFFERED, READ ANSWERS AND ALLOW FOR MULTIPLE RESPONSES.*		**YES**	**NO**	**N/A**
		A. Oral contraceptive	1	0	98
		B. Injectable/Depo	1	0	98
		C. Condoms	1	0	98
		D. IUD	1	0	98
		E. Implant	1	0	98

		F. Female sterilization	1	0	98
		G. Male sterilization	1	0	98
		H. Fertility awareness methods	1	0	98
		I. Emergency contraception	1	0	98
		J. Other ____________________ *SPECIFY*	1	0	98
**Q119**	What type of abortion services do you currently offer at this clinic?<br><br>*READ ANSWERS AND ALLOW FOR MULTIPLE RESPONSES.*		**YES**	**NO**	
		A. Medication Abortion	1	0	
		B. Manual Vacuum Aspiration (MVA)	1	0	
		C. Dilation and Evacuation	1	0	
**Q120**	What type of abortion services do you currently offer at this clinic?<br><br>*CHECK ANSWERS TO QUESTION Q119. IF SERVICES ARE NOT OFFERED, MARK N/A. FOR SERVICES OFFERED, READ ANSWERS AND ALLOW FOR MULTIPLE RESPONSES.*		**YES**	**NO**	**N/A**
		A. Medication Abortion	1	0	98
		B. Manual Vacuum Aspiration (MVA)	1	0	98
		C. Dilation and Evacuation	1	0	98

**Q121**	How many clients do you see at this facility in a typical week (average or estimate)?	|__|__|__| clients
**Q122**	How many clients do you see at this facility for family planning services in a typical week (average or estimate)?	|__|__|__| clients
**Q123**	How many clients do you see at this facility for abortion services in a typical **month** (average or estimate)?	|__|__|__| clients

PROVIDER TRAINING AND PRACTICE WITH IUD

READ TO PROVIDER: ***Now, I will ask you about your experience with the IUD, such as any training you've received and how frequently you insert or remove the device.***

TRAINING FREQUENCY

NO.	QUESTIONS	RESPONSES	CODE	SKIP
Q201	Have you ever been trained to insert an IUD?	Yes<br>No	1<br>0	 → Q208
Q202	How long ago was your most recent training related to IUDs?<br><br>*IF CLIENT ANSWERS LESS THAN 1 YEAR. WRITE 00 IN A*	A. |__|__| years<br>B. |__|__| months		
Q203	When were you first trained to insert an IUD? (If the only training was one mentioned above, report the same time period.)<br><br>*IF CLIENT PROVIDES THE SAME RESPONSE AS QUESTION Q202, RECORD THE SAME RESPONSE HERE. IF CLIENT ANSWERS LESS THAN 1 YEAR, WRITE 00 IN A AND USE B TO FILL IN THE NUMBER OF MONTHS.*	A. |__|__| years<br>B. |__|__| months		
Q204	At what stage(s) of your professional career have you received training on IUDs?<br><br>*READ ANSWERS AND ALLOW FOR MULTIPLE RESPONSES.*	**YES NO**<br>A. Medical/nursing Student 1 0<br>B. Resident 1 0<br>C. As part of previous job (at another facility) 1 0<br>D. As part of current job (while at this facility) 1 0		

TRAINING COMPONENTS

NO.	QUESTIONS	RESPONSES	YES	NO	DON'T KNOW	SKIP
Q205	Please tell me which of these components was a part of your most recent IUD training. If you don't remember, please tell me.<br><br>*READ ANSWERS AND ALLOW FOR MULTIPLE RESPONSES. MARK N/A IF CLIENT DOES NOT REMEMBER.*	A. Live classroom lecture	1	0	98	
		B. Video lecture	1	0	98	
		C. I observed an insertion on a pelvic model	1	0	98	
		D. I observed an insertion on a live patient	1	0	98	
		E. I practiced an insertion on a pelvic model during training	1	0	98	
		F. I practiced an insertion on a live patient during training	1	0	98	
		G. I practiced a removal on a pelvic model during training	1	0	98	

		H. I practiced a removal on a live patient during training	1	0	98	
		I. After the training, a trainer came to my facility and observed me doing an insertion on a live patient	1	0	98	
		J. After the training, a trainer came to my facility and observed me doing an insertion on a pelvic model	1	0	98	
Q206	Which organization provided your most recent IUD training? If you don't remember, please tell me.<br><br>*SELECT ONE.*	PSI			1	
		UNFPA			2	
		Ministry of Health			3	
		JHPIEGO			4	
		Don't Know			98	
		Other ___________________<br>*SPECIFY*			96	
Q207	After the most recent training ended, did you receive any of the following activities as post-training support? If you don't remember, please tell me.<br><br>*READ ANSWERS AND ALLOW FOR MULTIPLE RESPONSES. MARK N/A IF CLIENT DOES NOT REMEMBER.*		**YES**	**NO**	**DON'T KNOW**	
		A. A supervisory visit within 1 month of completing the training	1	0	98	
		B. A supervisory visit within 6 months of completing the training	1	0	98	
		C. A refresher training (small class or demonstration to review aspects of the training)	1	0	98	
		D. Opportunity to participate in an "event day" where IUDs were inserted free of charge to clients	1	0	98	
		E. Other ________________<br>*SPECIFY*	1	0	98	

IUD INSERTION FREQUENCY

NO.	QUESTIONS	RESPONSES	CODE	SKIP
Q208	Have you ever inserted an IUD?	Yes	1	
		No	0	→ End section
Q209	How long has it been since you last inserted an IUD?<br><br>*SELECT ONE.*	Within the last month	1	
		Between 1 and 6 months	2	
		Between 7 and 12 months	3	→ Q211
		More than 1 year ago	4	→ Q211
Q210	How many IUDs have you inserted in the last 6 months?	|__|__| IUDs		

**Q211**	How long has it been since you last removed an IUD?<br><br>*SELECT ONE.*	Within the last month<br><br>Between 1 and 6 months<br><br>Between 7 and 12 months<br><br>More than 1 year ago	1<br><br>2<br><br>3<br><br>4	 <br>→ End section<br><br>→ End section
**Q212**	How many IUDs have you removed in the last 6 months?	|__|__| IUDs		

PROVIDER KNOWLEDGE OF IUD

READ TO PROVIDER: ***Now, I would like to ask you some factual questions about the IUD. This is not a test, but this information will allow us to provide better information to you and other providers like you in the future.***

NO.	QUESTIONS	RESPONSES	CODE	SKIP
**Q301**	How effective is the widely used copper TCu 380A IUD at preventing pregnancy annually?<br><br>*SELECT ONE.*	Less than 85% effective<br>Between 85% an 90% effective<br>Between 90% and 95% effective<br>Greater than 99% effective	1<br>2<br>3<br>4	
**Q302A**	What is the maximum length of time a woman can use the TCu 380A IUD after it is inserted?<br><br>*SELECT ONE.*	2 years or less<br>3 years<br>5 years<br>6 years<br>7 years<br>10 years<br>12 years	1<br>2<br>3<br>4<br>5<br>6<br>7	
**Q302B**	What is the maximum length of time a woman can use the Multiload Cu 375 IUD after it is inserted?<br><br>*SELECT ONE.*	2 years or less<br>3 years<br>5 years<br>6 years<br>7 years<br>10 years<br>12 years	1<br>2<br>3<br>4<br>5<br>6<br>7	
**Q303**	What do researchers believe is the main mechanism of action of copper-bearing IUDs at preventing pregnancy?<br><br>*SELECT ONE.*	Changes in the woman's uterus that destroy a fertilized egg<br>Changes in the woman's uterus that prevent a fertilized egg from implanting<br>Preventing fertilization by reducing the number of sperm that reach the egg<br>Preventing fertilization by stopping the egg from being released by ovaries<br>None of the above	1<br>2<br>3<br>4<br>5	
**Q304**	True or False? When can the IUD be safely inserted, provided it is reasonably certain the woman is not pregnant?	**TRUE** / **FALSE**<br>A. When a woman is menstruating — 1 / 0		

	READ EACH STATEMENT AND RECORD TRUE OR FALSE FOR EACH ANSWER.	B. Anytime during the menstrual cycle, provided the woman is not pregnant and has no signs of infection.	1	0	
		C. Within 48 hours post-partum, provided there is no infection or hemorrhage	1	0	
		D. Up to 7 days post-partum, provided there is no infection or hemorrhage	1	0	
		E. Four weeks after delivery	1	0	
		F. 6 months after delivery	1	0	

PROVIDER KNOWLEDGE AND ATTITUDES TOWARD IUD APPROPRIATENESS AND RISK

KNOWLEDGE OF CLINICAL APPROPRIATENESS

READ TO PROVIDER: ***I will now describe women with certain clinical characteristics. In your opinion, please tell me if the woman is medically eligible for a copper IUD , if she is eligible, but with some screening or caution, or if she is not medically eligible for this method. Assume the woman is otherwise healthy.***

NO.	QUESTION	RESPONSE		
Q401		MEDICALLY ELIGIBLE	ELIGIBLE WITH SCREENING	NOT ELIGIBLE
	A. Currently breastfeeding	1	2	3
	B. HIV positive	1	2	3
	C. Recently undergone first trimester abortion	1	2	3
	D. History of ectopic pregnancy	1	2	3
	E. Smokes < 15 cigarettes per day	1	2	3
	F. Overweight/obese	1	2	3
	G. Hypertension patient	1	2	3
	H. On Anti-Retroviral Therapy (clinically well)	1	2	3
	I. Vaginal discharge	1	2	3
	J. Had pelvic inflammatory disease 3 years ago	1	2	3
	K. Current STI patient	1	2	3
	L. Iron-deficiency anemia	1	2	3
	M. Irregular menstrual pattern	1	2	3
	N. Less than 48 hours post-partum	1	2	3

ATTITUDE TOWARD DEMOGRAPHIC APPROPRIATENESS

READ TO PROVIDER:

I will now describe a brief characteristic about a woman. Assume this woman came to your clinic and wanted you to help her choose a family planning method. She is otherwise healthy. Please tell me if you would recommend the IUD to her as a method, would not recommend the IUD, or don't know/are not sure.

NO.	QUESTION	RESPONSE		
Q402		RECOMMEND	NOT RECOMMEND	NOT SURE
	A. A woman who is not married	1	2	98
	B. A woman who has no children (nulliparous)	1	2	98
	C. A woman who is 17 years old	1	2	98
	D. A woman who has more than one sexual partner	1	2	98

	E. A woman who has one child	1	2	98
	F. A woman who is of very small stature (short, tiny, etc.)	1	2	98
	G. A woman who wants to delay her next pregnancy	1	2	98
	H. A woman who is illiterate	1	2	98
	I. A woman who has 4 children	1	2	98
	J. A woman who does not want to have any more children	1	2	98
	K. A woman whose sexual partner is not monogamous	1	2	98
	L. A woman who does heavy physical labor every day	1	2	98
	M. A woman who is very poor	1	2	98

ATTITUDE TOWARD SIDE EFFECTS

NO.	QUESTION	RESPONSE			
Q403	In your opinion, what are the side effects or adverse outcomes associated with using a copper IUD?<br><br>*DO NOT PROMPT. ALLOW CLIENT TO SPEAK FREELY, AND RECORD ANSWERS AS SHARED. OTHER RESPONSES SHOULD BE RECORDED UNDER "OTHER".*		**YES**	**NO**	
		A. Irregular spotting (bleeding)	1	0	
		B. Nausea / vomiting	1	0	
		C. Weight Gain	1	0	
		D. Headaches	1	0	
		E. Increased risk of cancer	1	0	
		F. Irregular menstruation	1	0	
		G. Excessive menstruation	1	0	
		H. Painful menstruation	1	0	
		I. Cramping/abdominal pain	1	0	
		J. Increased risk of acquiring HIV	1	0	
		K. Increased risk of acquiring other STI	1	0	
		L. Infertility	1	0	
		M. Delayed return of fertility after removal	1	0	
		N. Increased risk of ectopic pregnancy	1	0	
		O. Amenorrhea	1	0	
		P. Other ________________ *SPECIFY*	1	0	
		Q. Other ________________ *SPECIFY*	1	0	
Q404	Do you consider *[SIDE EFFECT]* to be unacceptable, preventing you from recommending the copper IUD?<br><br>*CHECK ANSWERS TO QUESTION Q403. IF SIDE EFFECT WAS NOT MENTIONED, MARK N/A.*		**YES**	**NO**	**N/A**
		A. Irregular spotting (bleeding)	1	0	98
		B. Nausea / vomiting	1	0	98
		C. Weight Gain	1	0	98

		D. Headaches	1	0	98
		E. Increased risk of cancer	1	0	98
		F. Irregular menstruation	1	0	98
		G. Excessive menstruation	1	0	98
		H. Painful menstruation	1	0	98
		I. Cramping/abdominal pain	1	0	98
		J. Increased risk of acquiring HIV	1	0	98
		K. Increased risk of acquiring other STI	1	0	98
		L. Infertility	1	0	98
		M. Delayed return of fertility after removal	1	0	98
		N. Increased risk of ectopic pregnancy	1	0	98
		O. Amenorrhea	1	0	98
		P. Other ________________ *SPECIFY*	1	0	98
		Q. Other ________________ *SPECIFY*	1	0	98

PROVIDER'S NON-CLINICAL PERCEPTIONS ABOUT IUD

PROMOTIVE FACTORS FOR IUD RECOMMENDATION

READ TO PROVIDER: ***Next, I would like to ask you about the reasons you do or do not recommend the IUD.***

CHECK QUESTION Q208. IF RESPONDENT HAS NOT PREVIOUSLY INSERTED AN IUD, SKIP QUESTION Q501 TO QUESTION Q502. IF RESPONSDENT HAS PREVIOUSLY INSERTED AN IUD, DO NOT PROMPT RESPONSES FOR QUESTIONS Q501. ALLOW RESPONDENT TO SPEAK FREELY, AND RECORD THEIR RESPONSE VERBATIM. THE POST CODE RESPONSES ARE FOUND BELOW.

**Q501**	You mentioned that you have experience in inserting IUDs. Could you please tell me what are the factors or characteristics of IUDs which lead you to recommend and provide IUDs to women?

____

____

____

____

NO.	RESPONSES	CODE YES	CODE NO	SKIP
**Q501**		**YES**	**NO**	
	A. It is a safe contraceptive	1	0	
	B. It is affordable for my clients	1	0	
	C. It is a long term method	1	0	
	D. It does not have many side effects	1	0	
	E. It is very effective at preventing pregnancy	1	0	
	F. It is easily reversible / return to fertility is rapid	1	0	
	G. Client requests the IUD	1	0	
	H. It is being promoted by [PSI]	1	0	
	I. It is being promoted by Ministry of Health	1	0	
	J. It is a profitable method for me	1	0	
	K. It is easy for women to use	1	0	
	L. It is hormone free	1	0	
	M. Other ________________________________ *SPECIFY*	1	0	

BARRIERS TO IUD RECOMMENDATION

IF QUESTION Q501 WAS SKIPPED, ASK THE CLIENT QUESTION Q502 AND END THE SECTION AFTERWARD.
IF QUESTION Q501 WAS ANSWERED, SKIP QUESTION Q502 TO QUESTION Q503.

DO NOT PROMPT RESPONSES FOR QUESTION Q502 OR Q503. ALLOW RESPONDENT TO SPEAK FREELY, AND RECORD THEIR RESPONSES VERBATIM. THE POST CODE RESPONSES ARE FOUND BELOW.

**Q502**	What are some of the reasons why you do not recommend or provide more IUDs to women then you currently do?
**Q503**	Could you please tell me what are the factors or characteristics of IUDs which prevent you from recommending or providing them to women?

____

____

____

____

**NO.**	**RESPONSES**	**CODE**		**SKIP**
**Q502/503**		**YES**	**NO**	
	A. Insertion procedure is complicated	1	0	
	B. I do not have adequate supply of IUD	1	0	
	C. I do not have the equipment required for IUD insertion	1	0	
	D. Requires disinfection of clinic/instruments	1	0	
	E. Requires helper (another staff person) for insertion	1	0	
	F. Takes too much time for counseling of women	1	0	
	G. Takes too much time for insertion	1	0	
	H. Clients do not ask for it	1	0	
	I. It is too expensive for my clients	1	0	
	J. It is not a profitable method for me	1	0	
	K. I am not comfortable inserting IUD	1	0	
	L. I need training in inserting IUD	1	0	
	M. There is the possibility of uterus perforation	1	0	
	N. The side effects are too much for the client	1	0	
	O. It is not a suitable method for most of my clients	1	0	
	P. It can be expelled	1	0	
	Q. Clients can get pregnant when using IUD	1	0	
	R. Clients have difficulty getting pregnant after using the IUD	1	0	
	S. Insertion is too painful for client	1	0	

	T. It can shift / be displaced in uterus	1	0	
	U. I am concerned I will acquire HIV during the insertion	1	0	
	V. Clients are afraid of IUD	1	0	
	W. Requires follow-up visit by client	1	0	
	X. Other ________________________ *SPECIFY*	1	0	

PROVIDER SELF-EFFICACY

READ TO PROVIDER: ***Last, I would like to ask if you agree or disagree with the following statements about the IUD. For the each statement, please respond from a scale of 1 to 5, where 1 means you strongly disagree and 5 means you strongly agree.***

NO.	QUESTION	RESPONSE					
		STRONGLY DISAGREE				STRONGLY AGREE	NO RESPONSE
Q601	I can insert the IUD with little pain to the client.	1	2	3	4	5	99
Q602	IUDs are too difficult to insert.	1	2	3	4	5	99
Q603	IUDs are too time consuming to insert.	1	2	3	4	5	99
Q604	The risk of complication when inserting an IUD is too great.	1	2	3	4	5	99
Q605	I would recommend the IUD to a friend or family member who is medically eligible.	1	2	3	4	5	99
Q606	When inserting the IUD, I worry about infecting myself with a sexually transmitted disease or HIV.	1	2	3	4	5	99
Q607	There are too many issues to consider when deciding if a woman can use an IUD.	1	2	3	4	5	99
Q608	I feel comfortable explaining IUD issues to my patients.	1	2	3	4	5	99
Q609	There are many days when I am too busy to discuss about/provide IUDs.	1	2	3	4	5	99
Q610	It is very difficult to convince clients that many rumors about the IUDs are actually false	1	2	3	4	5	99
Q611	If a woman came to me asking for an IUD, I would be happy to provide it	1	2	3	4	5	99
Q612	I feel comfortable that I can insert an IUD safely and effectively.	1	2	3	4	5	99
Q613	I feel comfortable that I can remove an IUD safely and effectively	1	2	3	4	5	99
Q614	Provision of IUDs is not profitable for me.	1	2	3	4	5	99
Q615	Providing IUD insertion services is a good use of my skills and experience.	1	2	3	4	5	99
Q616	I worry that complications arising from an IUD will damage the reputation of my clinic.	1	2	3	4	5	99

psi
