# Supplementary material for: Knowledge and perceptions of the intrauterine device among family planning providers in Nepal: a cross-sectional analysis by cadre and sector
Source: BMC Health Serv Res. 2015 Jan 28;15:39. doi: 10.1186/s12913-015-0701-y (PMC4322443; doi:10.1186/s12913-015-0701-y)
Supplement: Additional file 2: — Proportions and mean of provider sociodemographic characteristics & professional experience, by sector. [file 12913_2015_701_MOESM2_ESM.pdf]

Additional File 2   Proportions and mean of provider sociodemographic characteristics & professional experience by sector[1]

Variable	MSS franchise n = 176	Public sector n = 122	Private non-franchise n = 47	All sectors n = 345
Sociodemographic				
Age (mean)	28.9 ± 7.41	39.1 ± 8.59	32.4 ± 8.62***	33.0 ± 9.26
Have children	112 (63.6)	108 (88.5)	32 (68.1)***	252 (73.0)
Currently using contraception	68 (38.6)	64 (52.5)	22 (46.8)	154 (44.6)
Have used IUD	20 (11.4)	17 (13.9)	9 (19.2)	46 (13.3)
Are an ANM	154 (87.5)	94 (77.1)	28 (59.6)***	276 (80.0)
Facility-related				
Years employed at facility (mean)	4.1 ± 4.44	8.1 ± 6.87	6.4 ± 5.73***	5.8 ± 5.86
Owns facility	89 (50.6)	0 (0.0)	13 (27.7)***	102 (29.6)
Facility is primary place of work	141 (80.1)	104 (85.3)	42 (89.4)	287 (83.2)
Facility is located in hill region	96 (54.6)	39 (32.0)	9 (19.2)***	144 (41.7)
Number of FP clients per week (mean)	19.1 ± 17.48	32.8 ± 27.70	27.4 ± 20.49***	25.1 ± 22.84
Number of FP methods (mean)	6.0 ± 1.38	7.1 ± 1.37	6.7 ± 1.94***	6.5 ± 1.54
Facility offers IUDs	157 (89.2)	112 (91.8)	45 (95.7)	314 (91.0)
IUD experience				
Have inserted IUD < 6 months ago	168 (95.5)	98 (80.3)	42 (89.4)***	308 (89.3)
Number of IUDs inserted in last 6 months (mean)	31.1 ± 24.54	20.2 ± 37.82	23.8 ± 27.08***	26.3 ± 30.55
Years since most recent IUD training (mean)	0.8 ± 0.93	4.7 ± 4.88	3.6 ± 3.86***	2.6 ± 3.73

[1] Anova and chi-squared tests were used to test the differences between sectors. Differences are indicated by ** $p < 0.05$ and *** $p < 0.001$. Values are given as mean ± SD or number (percentage).
